# Socioeconomic disparities in breast cancer survival: relation to stage at diagnosis, treatment and race

**DOI:** 10.1186/1471-2407-9-364

**Published:** 2009-10-14

**Authors:** Xue Qin Yu

**Affiliations:** 1 Cancer Epidemiology Research Unit, Cancer Council New South Wales, PO Box 572, Kings Cross, NSW 1340, Australia

## Abstract

**Background:**

Previous studies have documented lower breast cancer survival among women with lower socioeconomic status (SES) in the United States. In this study, I examined the extent to which socioeconomic disparity in breast cancer survival was explained by stage at diagnosis, treatment, race and rural/urban residence using the Surveillance, Epidemiology, and End Results (SEER) data.

**Methods:**

Women diagnosed with breast cancer during 1998-2002 in the 13 SEER cancer registry areas were followed-up to the end of 2005. The association between an area-based measure of SES and cause-specific five-year survival was estimated using Cox regression models. Six models were used to assess the extent to which SES differences in survival were explained by clinical and demographical factors. The base model estimated the hazard ratio (HR) by SES only and then additional adjustments were made sequentially for: 1) age and year of diagnosis; 2) stage at diagnosis; 3) first course treatment; 4) race; and 5) rural/urban residence.

**Results:**

An inverse association was found between SES and risk of dying from breast cancer (p < 0.0001). As area-level SES falls, HR rises (1.00 → 1.05 → 1.23 → 1.31) with the two lowest SES groups having statistically higher HRs. This SES differential completely disappeared after full adjustment for clinical and demographical factors (p = 0.20).

**Conclusion:**

Stage at diagnosis, first course treatment and race explained most of the socioeconomic disparity in breast cancer survival. Targeted interventions to increase breast cancer screening and treatment coverage in patients with lower SES could reduce much of socioeconomic disparity.

## Background

Previous studies have documented substantial disparities in breast cancer survival in relation to socioeconomic status (SES) as measured either at the individual [1,2] or area levels [3-6] in the United States, with women in lower SES groups showing poorer survival. Factors that may mediate these disparities include differences in the stage at diagnosis, access to and quality of care delivered and other correlates of low SES [7]. Women with less education and those who are unemployed, reside in a poor area, or are uninsured or under-insured are more likely to be diagnosed at later stages [8-10], and are less likely to receive optimal cancer care [11,12]. Race has been reported to be related with breast cancer survival independently of SES [5,13].

Limitations of most prior US studies that have examined disparities in breast cancer survival by SES are that they considered only women with early stage diagnosis [4,14-17] or those over age 65 years [1,4,9,13,17-19]. Other studies involved patients at a single institution [2,14]. Therefore, they may not be representative of the entire population diagnosed with breast cancer. In this study, I examined the extent to which these disparities was explained by stage at diagnosis, first course treatment, race and rural/urban residence by taking account of these variables, from the Surveillance, Epidemiology, and End Results (SEER) database, simultaneously and including all women diagnosed with breast cancer.

## Methods

### Study population

Women aged 15 years or older and diagnosed with first primary invasive breast cancer (ICDO-3 code: C50) [20] between January 1, 1998 and December 31, 2002 were identified through the 13 population-based cancer registries in the United States that participated in the SEER program (Atlanta, Connecticut, Detroit, Hawaii, Iowa, New Mexico, San Francisco-Oakland, Seattle-Puget Sound, Utah, Los Angeles, San Jose-Monterey, Rural Georgia and the Alaska Native Registry). Of the 113,905 women, a total of 1362 (1.2%) cases were excluded from the analysis because they were diagnosed at autopsy or through death certificate only (n = 705), had unknown race (n = 647) or missing residential address at diagnosis (n = 10).

### Study variables

The outcome variable was survival time after diagnosis of breast cancer. The primary study variable was a composite measure of SES. As the SEER program does not collect individual level measures of SES, a composite variable was used based on two characteristics in county of residence: "percent of adults with < 12 year education" and "percent of families living below the federal poverty line". Data were obtained from the 1990 U.S. Census. Educational level was categorized into 4 similar size groups (1 → 4 from high to low) and poverty rate was divided into 3 major groups using the cutpoints: ≤ 9.9% (low), 10-19.9% (medium), ≥ 20% (high) as recommended by others based on empirical research [21]. Counties were divided into four groups according to their levels of these two SES measures so that each group had similar number of cases. Counties with the educational level one (highest) and lowest poverty rate was categorized as high SES; counties with either educational level one and medium poverty rate, or lowest poverty rate and educational level two were assigned to the upper middle SES group; counties with educational level four and medium or high poverty rate, or educational level three and high poverty rate were classified as the lowest SES group; the remaining combination was the lower middle SES group. Women were allocated into each of the SES groups according to the county they lived in at diagnosis.

The following factors that may affect survival between SES groups were included in the analysis. Year of diagnosis were 1998 to 2002. Age at diagnosis was categorized into 5 groups: (15-44 years, 45-54 years, 55-64 years, 65-74 years and > = 75 years). Race was categorized into three broad groups (White, Black and other). Rural/urban residence was defined based on the rural-urban continuum codes for 2003 available at http://seer.cancer.gov/seerstat/variables/countyattribs/ruralurban.html. Women were categorized as living in an urban area if their county was located in a metro area (code 1, 2, 3 on the continuum codes). Stage at diagnosis, using American Joint Committee on Cancer (AJCC) stage [22], was categorized into 5 groups: stage I, II, III, IV and unknown stage or not applicable. Number of lymph nodes positive for those having lymph nodes examined was categorized into 3 groups: none, 1-3, and ≥ 4 [23]. Information on the first course treatment (surgery and/or radiation) was dichotomized into receipt/no receipt categories. Detailed surgery definition from SEER data can be found from SEER website http://seer.cancer.gov/manuals/historic/AppendC.pdf. Briefly, there are two types of surgeries: breast-conserving surgery and mastectomy. Breast-conserving surgery was defined as receiving segmental mastectomy, lumpectomy, nipple resection, excisional biopsy or partial mastectomy unspecified, and mastectomy included total, modified radical, radical, extended radical mastectomy or mastectomy unspecified. Chemotherapy, immunotherapy and hormonal therapy were not considered since they are not in the SEER public-use files.

### Statistical analysis

The SEER data provide vital status and survival time for each patient, calculated in months using date of diagnosis and end of study, either date of death or the end of 2005 (the cut-off date of follow-up), whichever occurred first. Cause-specific survival was used for the hazard ratios (HR) estimation. A HR represents the risk of dying from breast cancer. Women were censored for death from causes other than breast cancer, or at the end of 2005.

In order to control for the effect of multiple factors simultaneously on disparities in breast cancer survival, the effect of SES on survival was estimated using Cox proportional hazard models. Briefly, these analyses consisted of six models. The basis model (model 0) estimated HR by SES without any adjustment using the highest SES group as a reference. Model 1 adjusted for age group at diagnosis and year of diagnosis. Ninety-five percent confidence intervals (CIs) for the HRs were calculated using the estimated coefficients and standard errors from the Cox regression models. A test of linear hypotheses about the effect of SES with p-value of < 0.05 was considered to be statistically significant.

In models two to five, HR for each SES group was estimated with additional adjustment for (2) AJCC stage and number of positive lymph nodes, (3) first course treatments, (4) race, (5) rural/urban residence at diagnosis, respectively. This was used to ascertain if adjustment for each group of factors reduced the survival difference between SES groups [7,24]. The validity of the proportional hazards of the predictors was tested by stratifying on the predictors and comparing the parameter estimates of the stratified model with those from the model including the variable as a proportional predictor [25,26]. No violations of proportionality of hazard were found. Patient data were obtained using the SEER software SEER*Stat version 6.4.4. All statistical analyses were performed using SAS 9.1 (SAS Institute, NC).

## Results

A total of 112,543 women diagnosed with breast cancer were included in this analysis. Univariate analysis showed that all the variables listed in Table 1 were highly associated with SES level based on area of residence at the time of diagnosis. As shown in Table 1, women living in the lowest SES areas had the lowest percentage of early stage (I): 41.4% vs 45.7-46.7%, and highest percentage of advanced stages (III, IV): 13.0% vs 9.5-9.9% and were more likely to have ≥ 4 lymph nodes positive: 16.0% vs 12.3-12.8%. The proportion of Black women living in the lowest SES areas was nearly four times higher than that of the highest SES areas: 16.1% vs 4.3%. The proportions of women from the two lowest SES areas who received the first course treatment were lower than that of the highest SES areas, especially for radiation (<48% vs 57.3%). The proportion living in rural areas for women from lower middle group was much higher than these of other groups (23.1% vs <4.0%).

**Table 1:** Distribution of study variables by socioeconomic status (SES), breast cancer diagnosed in 1998-2002

		Percentage of area-based SES group				
			
	Number of cases	Highest SES	Upper middle	Lower middle	Lowest SES	p-value
Age at diagnosis (year)						<0.0001
15-44	15520	14.4	14.0	12.4	14.4	
45-54	26545	25.2	23.3	22.3	23.6	
55-64	25545	23.3	23.2	21.9	22.5	
65-74	22462	18.6	19.7	21.1	20.3	
75+	22471	18.4	19.8	22.3	19.2	
Year of diagnosis						<0.0001
1998	22134	19.4	18.4	21.4	19.4	
1999	22487	19.5	18.7	21.3	20.1	
2000	22385	20.2	21.0	18.6	20.0	
2001	22791	20.3	20.9	19.6	20.2	
2002	22746	20.6	21.0	19.1	20.3	
Race						<0.0001
White	92717	90.8	79.7	85.4	74.4	
Black	9831	4.3	7.2	6.3	16.1	
Other	9995	4.9	13.1	8.3	9.5	
Rural/urban residence						<0.0001
Rural	9819	2.7	4.0	23.1	4.0	
Urban	102724	97.3	96.0	76.9	96.0	
AJCC^† ^stage						<0.0001
I	50608	46.7	45.7	46.6	41.4	
II	41646	36.2	37.1	35.1	39.5	
III	7188	5.7	5.8	5.5	8.3	
IV	4770	3.8	4.1	4.3	4.7	
Unknown	8331	7.6	7.3	8.5	6.2	
Number of positive lymph nodes						<0.0001
None	60700	64.9	64.0	65.4	60.2	
1-3	21948	22.8	23.2	21.9	23.9	
≥ 4	12896	12.3	12.8	12.7	16.0	
First course treatment received						
Surgery	106076	95.3	94.5	93.6	93.8	<0.0001
Radiation	58144	57.3	55.1	47.8	47.7	<0.0001

† AJCC: American Joint Committee on Cancer.

Table 2 shows the results of Cox regression modelling with an inverse association between SES and risk of dying from breast cancer (1.00 → 1.05 → 1.23 → 1.31). When adjusting for age at diagnosis and calendar year (model 1), the HRs remained significantly higher for women residing in the two lowest SES areas; women from the lowest SES areas had 19% higher hazard of cancer-related death (HR = 1.19) than women from the reference - highest SES group (p < 0.0001). After additional adjustment for stage at diagnosis (model 2), the HR for the lowest SES group dropped to 1.10, but remained significant. Further adjustment for first course treatment (model 3) reduced the HRs for the lowest SES groups slightly (1.10 → 1.08). Then when adding race to the model (model 4), the HR for the lowest SES group was non-significant (HR = 1.03, 95% CI: 0.97-1.08). Further, the overall effect of SES was reduced from highly significant (p < 0.0001) in the base model to non-significant (p = 0.07) in model 4. Final adjustment for rural/urban residence (model 5) further weakened this SES differential (p = 0.20) with no SES group having significant higher HR than that of the top SES group.

**Table 2:** Hazard ratio (HR) of cancer-specific mortality from breast cancer by socioeconomic status (SES)

	Model 0	Model 1	Model 2	Model 3	Model 4	Model 5
	
Area-based SES group	SES only	Model 0 + age & year	Model 1 + stage	Model 2 + treatment	Model 3 + race	Model 4 + rural/urban
	
	HR & (CI†)	HR & (CI)	HR & (CI)	HR & (CI)	HR & (CI)	HR & (CI)
Highest	1.00	1.00	1.00	1.00	1.00	1.00
Upper middle	1.05 (0.99-1.11)	1.01 (0.96-1.07)	1.00 (0.94-1.06)	1.00 (0.95-1.06)	0.99 (0.93-1.05)	0.99 (0.93-1.05)
Lower middle	1.23 (1.16-1.29)	1.09 (1.03-1.15)	1.08 (1.03-1.14)	1.07 (1.01-1.12)	1.06 (1.00-1.12)	1.04 (0.99-1.10)
Lowest	1.31 (1.24-1.38)	1.19 (1.13-1.26)	1.10 (1.04-1.16)	1.08 (1.03-1.14)	1.03 (0.97-1.08)	1.03 (0.97-1.08)

p-value for SES	<0.0001	<0.0001	<0.0001	0.003	0.07	0.20

† 95% confidence intervals.

**Figure 1:**
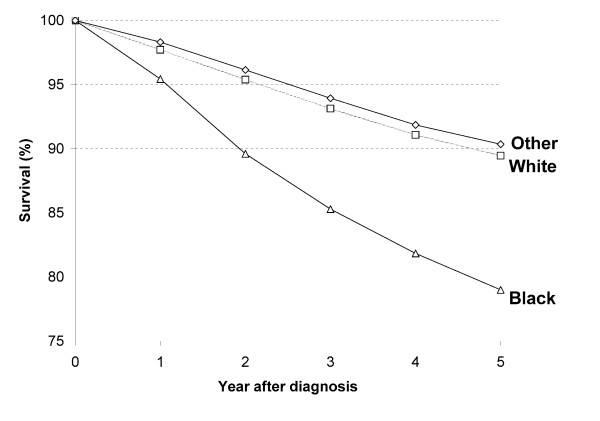
**Cause-specific survival from breast cancer diagnosed 1998-2002 followed-up to 2005 by race**.

Race-specific survivals were presented in Figure 1.

## Discussion

An inverse association was found between SES and risk of dying from breast cancer among 112,543 breast cancer patients. More importantly, stepwise adjustment for stage at diagnosis, first course treatment, race and rural/urban residence completely eliminated the survival disparity associated with lower SES. The strength of this study is that it considered women of all ages diagnosed with any stage of breast cancer in SEER database and thus had much broader coverage and greater statistical power than previous studies of this issue.

A strength of this study is that the finding of a social association in breast cancer survival was not affected by choice of data source (17 or 13 SEER registries) or categories of SES (quintile or quartile, single or composite). This implies that the models are robust, and the effect of place of residence was probably real and not due to statistical artifact. These results were also consistent with many other studies examining SES disparities in breast cancer survival in the United States [1,3] and other parts of the world [7,27]. Further this study is population-based and includes all women diagnosed with breast cancer in the 13 SEER areas from 1998 to 2002 and followed-up until the most recent cut-off date - December 31, 2005. Therefore, these results potentially reflected experiences of the entire population diagnosed with breast cancer in the 13 SEER areas and provided a full picture of most recent socioeconomic disparity in breast cancer survival.

This analysis is limited by the allocation of cases to SES groups using aggregated data. It is possible that individual people may have been misclassified and the inferences at the area level do not directly transfer to individuals. However, several studies have demonstrated the importance of area-based socioeconomic measures in measuring health inequality in the United States [28] and other parts of the world [7,27]. The second limitation of this study is the quality and completeness of treatment data in the SEER database: adjustment for the first course treatment (surgery and radiation) would not necessarily control for all dimensions of treatment for breast cancer, and in addition chemotherapy and hormonal therapy data are not available in the SEER public-use files. The data was also analyzed after categorizing receipt of treatment according to stage at diagnosis, surgery (mastectomy or breast conserving surgery) and radiation; and it was found that this grouping did not change the main findings.

Stage at diagnosis explained a large part of the SES disparity in breast cancer survival. This is likely because women living in the lowest SES areas had highest percent of advanced stages (III, IV) and ≥ 4 lymph nodes positive (Table 1) and much worse survival for those with advanced stage disease. This unfavorable stage distribution for women from the lowest SES areas was likely caused by lower mammography rates. Lack of health insurance and lower financial resources are known to be associated with lower mammography rates [8-10,29,30] and lack of, or delayed follow-up after an abnormal mammogram [31].

The contribution of first course treatment to the SES differential in breast cancer survival was rather small in this study because a large part of this differential had been captured by the differences in stage distribution between SES groups; those women diagnosed with later stage disease are more likely to receive inadequate treatment. However, possible explanations for women residing in lower SES areas for receiving inadequate care more often include lack of health insurance [14], comorbidities [32] and patients refusal or not adherence to therapies [2,33], and provider bias [11,34].

The gap in hazard of dying from breast cancer between the highest and lowest areas was reduced further and the overall effect of SES became non-significant (p = 0.07) after additional adjustment for race (Table 2). This is in part because blacks were disproportionately represented in the lowest SES group (16.1%) (Table 1) and had lower survival rate (Figure 1). However, the contribution of race to the SES differential in breast cancer survival was modest because a substantial proportion of SES disparities associated with race may have been captured by differences in stage at diagnosis and treatment. Consistent with the wide literature [3-5,13,18,19,33,35-39], the data indicated that black women were more likely to have later stages (III, IV) disease diagnosis (17.1% vs 10.6%) and less likely to receive first course treatment - surgery (90.3% vs 94.3%) and radiation (46.0% vs 51.7%) than the general population (data not shown). In addition, black women are more likely to have unfavorable tumor characteristics - negative hormone receptor status (ER/PR) or HER2-negative, higher-grade tumors and being diagnosed at younger age. Lower SES and inadequate access to medical care may interact with biological factors consequently leading to the disproportionate number of diagnoses of tumors with these unfavorable characteristics in younger black women. A more recent study found that black race was associated with increased mortality from breast cancer after adjusting for stage of disease and treatment, the authors therefore thought that biological or host genetic factors may be the potential source of the survival gap [40]. However, other studies reported that black women still had poorer outcomes from breast cancer after controlling for biological factors [1,4,37]. Overall, these data showed that race picks up some residual effect after controlling for measured variation in stage at diagnosis and first course treatment, which may be due to measurement errors, and some factors not related to these two variables, such as, biological characteristics. However, race is a complex and composite measure of many factors related with breast cancer survival and this study has limited ability to separate out these multiple dimensions of race that may influence survival.

There were some residual survival differentials between the highest and lower middle groups after adjusting for stage, first course treatment and race. This may be due to difference in the use of chemotherapy and/or hormonal therapy, which are not available in the SEER public-use data. Several studies found that women living in non-metropolitan areas in the U.S. were more likely to have delayed initiation of radiotherapy [32] and chemotherapy [17] after breast cancer surgery. The results of the HR for lower middle group becoming non-significant after further adjustment for rural/urban residence (Table 2), together with much more women in this group living in rural areas (Table 1), suggested that factors related to access to and/or time waiting for chemotherapy and/or radiotherapy may partly be attributable to the residual survival difference.

## Conclusion

Stage at diagnosis, first course treatment and race explained most of the socioeconomic disparities in breast cancer survival. Thus, targeted interventions to increase breast cancer screening and treatment coverage in patients with lower SES could reduce much of socioeconomic disparity in breast cancer survival.

## List of abbreviations

SEER: Surveillance, Epidemiology, and End Results; SES: socioeconomic status; AJCC: American Joint Committee on Cancer; HR: hazard ratio; CI: confidence intervals.

## Competing interests

The author declares that he has no competing interests.

## Authors' contributions

I conceived the study, did the data analysis, drafted the manuscript, and approved the final version submitted for publication.

## Pre-publication history

The pre-publication history for this paper can be accessed here:

http://www.biomedcentral.com/1471-2407/9/364/prepub
